# Fel d 1 specific IgE measurement for dog exclusive owners co-sensitized to dog and cat^☆^

**DOI:** 10.1016/j.waojou.2024.101007

**Published:** 2024-12-02

**Authors:** Lin Liang, Ah-Reum Hwang, Yoon Ji Shin, Kyoung Yong Jeong, Kyung Hee Park, Jae-Hyun Lee, Jung-Won Park

**Affiliations:** a Graduate School of Medicine, Yonsei University College of Medicine, Seoul, South Korea; b Institute of Allergy, Yonsei University College of Medicine, Seoul, South Korea; c Department of Internal Medicine, Yonsei University College of Medicine, Seoul, South Korea

**Keywords:** Cat, Cross-reactivity, Dog, Fel d 1

## Abstract

**Background:**

The diagnosis of the culprit allergen depends on exposure, symptoms at exposure, and the presence of specific IgE (sIgE). Pet allergens are sticky and can sensitize individuals without adoption history. Exclusive dog owners frequently exhibit both dog (e5) and cat dander sIgE (e1). We assessed whether the measurement of Fel d 1 sIgE (e94) can discriminate true cat sensitization from false positivity by cross-reactivity in the exclusive dog owners.

**Methods:**

Thirty-one patients with respiratory allergies who exclusively owned dogs were enrolled for this study. e5, e1, and e94 were measured with ImmunoCAP. ELISA inhibition was performed to assess cross-reactivity.

**Results:**

About 81% of patients (25/31) were both e5 and e1 positive, and 8 were also positive for e94. In the e94 positive, cat dander exhibited higher maximum inhibition of cat sIgE (94% vs 88%) and demonstrated lower IC_50_ (6.5 vs 737.9 BAU/mL) compared to dog dander. Conversely, in the e94 negative, dog dander demonstrated higher maximum inhibition of cat dander sIgE (71.9% vs 56.2%) and lower IC_50_ (172 vs 1947 BAU/mL) compared to cat dander. In the e94 positive, dog dander exhibited higher maximal inhibition for dog sIgE (91.5 vs 76.1%) and lower IC_50_ (10.6 vs 1679 BAU/mL) compared to cat dander, whereas in the e94 negative, the IC_50_ for cat dander could not be determined.

**Conclusions:**

Genuine co-sensitization to cats is notable even in individuals who exclusively own dogs. Positive e94 results could discriminate authentic cat sensitization from false positivity by cross-reactivity in these patients, underscoring the importance of comprehensive allergy assessment.

## Introduction

Allergic diseases caused by pets affect 10%–20% of the global population, presenting an increasingly significant public health concern.^1^^,^^2^ The growing trend of pet adoption further complicates this issue. Pet allergies are notable for their association with asthma exacerbations, decreased lung function, heightened airway inflammation, airway hyper-responsiveness, and increased use of inhaled corticosteroids (ICS), as demonstrated in numerous studies.^3^, ^4^, ^5^ While avoidance is an effective strategy, the majority of pet owners choose not to pursue this option. For these patients, allergen specific immunotherapy (AIT) represents the appropriate treatment approach, necessitating the precise identification of the responsible allergen.

For the diagnosis of the culprit allergen, 3 aspects should be considered. First, patients should be exposed to the suspected allergens. Second, patients develop allergic symptoms upon exposure. Third, the patients should have sIgE to the suspected allergens. However, diagnosing pet allergies is more complex. Pet allergens are adhesive and can sensitize individuals without a history of pet ownership, and cross-reactivity between cat and dog dander allergens is well recognized. Consequently, in real-world situations, pet allergy patients who exclusively own dogs frequently exhibit both dog and cat dander sIgE, complicating the diagnosis of the culprit allergen. Prevailing diagnostic strategies based on total dander extracts have significant drawbacks, including substantial fluctuations in allergen concentrations within the extracts^6^ and a high incidence of false positive results with uncertain clinical implications, which are attributed to the cross-reactivity of pet allergens from various furry animals.^7^ Therefore, molecular diagnosis of sIgE could contribute more accurate diagnosis of culprit allergen.^8^

The majority of pet allergens are classified into 5 distinct groups: lipocalins, secretoglobulins, serum albumins, kallikrein, and latherins. Among these, lipocalins and serum albumins are known to exhibit cross-reactivity with their counterparts across various mammalian species.^9^, ^10^, ^11^

The most dominant major allergen in cats is Fel d 1, and sIgE (e94) is detected in 95% of cat allergy patients. Fel d 1, a member of the secretoglobin family, is recognized for its lack of cross-reactivity with dog dander allergens. However, this issue is controversial, and recently, a Fel d 1-like allergen from dog dander was identified.^12^ Therefore, it remains uncertain whether a positive Fel d 1 sIgE result can exclude false positivity due to cross-reactivity in exclusive dog owners.

Other categories of cat dander allergens include lipocalins (Fel d 4, Fel d 7), serum albumin (Fel d 2), and IgA immunoglobulin containing alpha-gal (Fel d 5). These allergens exhibit cross-reactivity with corresponding dog dander allergens. Dog dander allergens consist of lipocalins (Can f 1, Can f 2, Can f 4, Can f 6), serum albumin (Can f 3), and prostatic kallikrein (Can f 5).^13^, ^14^, ^15^ Among the lipocalins, Can f 4 does not show cross-reactivity with lipocalins from other animal species.^9^^,^^16^

Given the frequent co-sensitization to cat and dog allergens among exclusive dog owners, accurately diagnosing true cat sensitization versus cross-reactivity is crucial for the appropriate prescription of allergen-specific immunotheray (AIT). In this study, we assess whether measuring Fel d 1 sIgE (e94) can solve this issue in allergy patients who exclusively own dogs.

## Methods

### Study population

Participants in this study were selected from individuals attending our outpatient clinic for suspected respiratory allergies, who underwent standard skin prick testing (SPT) with various inhaled allergens and assessment of e5, e1, and e94 in serum with ImmunoCAP assay.

All 31 recruited subjects reported experiencing allergic reactions upon contact with dogs. Allergic symptoms following exposure to dogs, based on self-reported assessments by participants, included rhino-conjunctivitis, respiratory difficulties, asthma, dermal reactions upon contact, and sneezing episodes. All participants owned dogs exclusively and did not have experience of cat cohabitation. Approval for the study was obtained from the Institutional Review Boards of Yonsei University Health System (Approval no. 4-2013-0397). All participants provided written consent before enrollment.

### SPT and serum specific IgE measurement

SPTs were performed on the dorsal regions of participants using a 26-gauge needle by an experienced operator. The allergens used included commercial skin prick test reagents (Hollister-Stier, Spokane, WA, USA); histamine solution (1.7 mg/mL in 0.3% phenol saline; Allergopharma, Reinbek, Germany) served as the positive control, and saline as the negative control. Wheal and flare responses induced by the allergens and histamine were evaluated 15 min after application. An SPT result was considered positive if the wheal diameter was ≥3 mm, while the negative control showed no reaction. In addition to SPTs, serum-specific immunoglobulin E (sIgE) levels against allergens e5, e1, and e94 were quantified using the ImmunoCAP assay (Thermo Fisher Scientific, Waltham, MA, USA), with serum samples stored at −20 °C until analysis. sIgE antibody concentrations were expressed in kU/L, and values equal to or exceeding 0.35 kU/L were considered positive.

### ELISA inhibition assays

Dog and cat dander extracts (10,000 BAU/mL, Hollister-Stier, Spokane, WA, USA) in sodium carbonate buffer, pH 9.6 were immobilized on a microplate (Corning Inc., Corning, NY, USA). Following blockage with 3% skim milk, the microplate underwent overnight incubation with pooled serum samples (diluted at 1:4). Samples were pre-treated with various concentrations of inhibitors (ranging from 0.64 to 10,000 BAU/mL) before introduction into each well, followed by a 1-h incubation period. IgE antibodies were detected by adding biotinylated goat anti-human IgE (dilution of 1:1000) (Vector, Burlingame, CA, USA), followed by a streptavidin-peroxidase conjugated antibody (dilution of 1: 1000) (Sigma-Aldrich). Color development commenced upon the addition of 3,3′5,5′-tetramethyl-benzidine substrate (Kirkegaard & Perry Laboratories, Gaithersburg, MD, USA). Absorbance readings at 450 nm were obtained after quenching the enzymatic reaction with 0.5 M H_2_SO_4_. The inhibition percentage was calculated using the formula: (1 − absorbance with inhibitors/absorbance without inhibitors) × 100.

## Results

### Characteristics of dog-allergic patients

Table 1 presents the demographic characteristics of the enrolled patients. All participants tested positive for e5, whereas 25 tested positive for e1. Among those positive for e1, only 8 patients were also positive for e94. Furthermore, 21 of the 31 patients underwent SPT, with 18 showing a positive reaction to dog dander extract and 15 demonstrating sensitivity to cat dander. In addition to allergic symptoms triggered by dog exposure, asthma and rhinitis were the most common symptoms, followed by conjunctivitis, chronic urticaria, and atopic dermatitis.

**Table 1:** Clinical features of enrolled dog allergy patients.

	No	Age	Sex	Symptoms upon contact with dog	SPT wheel diameter (mm)		e5 slgE (kU/L)	e1 slgE (kU/L)	Fel d 1 slgE (kU/L)
					Cat	Dog			
	1	27	M	R, U	6	4.5	27.1	3.24	2.05
	2	38	F	AS, R	9	5	>100	90.2	74.3
e1+/e94+	3	53	M	AS, R	4	5	>100	8.29	1.59
	4	33	M	AS, R, C	ND	ND	21.7	22.1	22.8
	5	31	M	AS, R	4	4	13	0.78	0.60
	6	21	M	AS, R	5	4	30.6	1.15	1.00
	7	36	M	R, U	ND	ND	22.6	0.59	8.73
	8	65	M	AS, R	ND	ND	24.2	2.84	1.55

	9	62	F	AS, R, C	ND	ND	8.49	0.68	0.03
	10	33	F	AD	ND	ND	7.87	0.40	0.00
	11	26	M	AS, R,	2	5	67.6	3.03	0.00
	12	30	F	AS, R, C	4.5	4	11.9	1.38	0.00
	13	28	F	AS, R	9	5	10.2	1.51	0.15
	14	27	M	AS, R	5	7.5	8.74	0.45	0.00
e1+/e94-	15	21	F	AS, R	0	5	23.1	1.28	0.28
	16	26	F	R, C	9	6.5	32.4	8.73	0.00
	17	57	F	AS, R	1	1.5	76.4	0.66	0.00
	18	34	M	AS	ND	ND	16.7	0.94	0.13
	19	30	F	R	7	4	2.34	10.5	0.00
	20	26	M	R	7.5	6	24.5	3.46	0.02
	21	27	F	AS, R, C	7.5	8	>100	20.7	0.03
	22	61	F	AS, R	0	6	18.9	0.41	0.33
	23	38	M	AS, R	6	6	56.6	2.71	0.00
	24	41	F	AS, R	7	0	2.89	0.60	0.05
	25	36	F	AS, R, C	ND	ND	4.61	0.80	0.00

	26	29	F	AS, R	ND	ND	3.38	0.24	0.00
	27	48	F	AS, R	2	3	3.6	0.05	0.00
e1-/e94-	28	48	M	U	ND	ND	6.41	0.12	0.33
	29	51	F	R, U	4	4	14.3	0.21	0.00
	30	53	F	AS, R	ND	ND	9.05	0.27	0.28
	31	16	F	AS, R	0	0	0.58	0.03	0.00

R, rhinitis; C, conjunctivitis; U, chronic urticarial; AS, asthma; AD, atopic dermatitis; ND: not done

### ELISA inhibition assays for evaluation of the cross-reactivity of the dog and cat dander

**Fig. 1:**
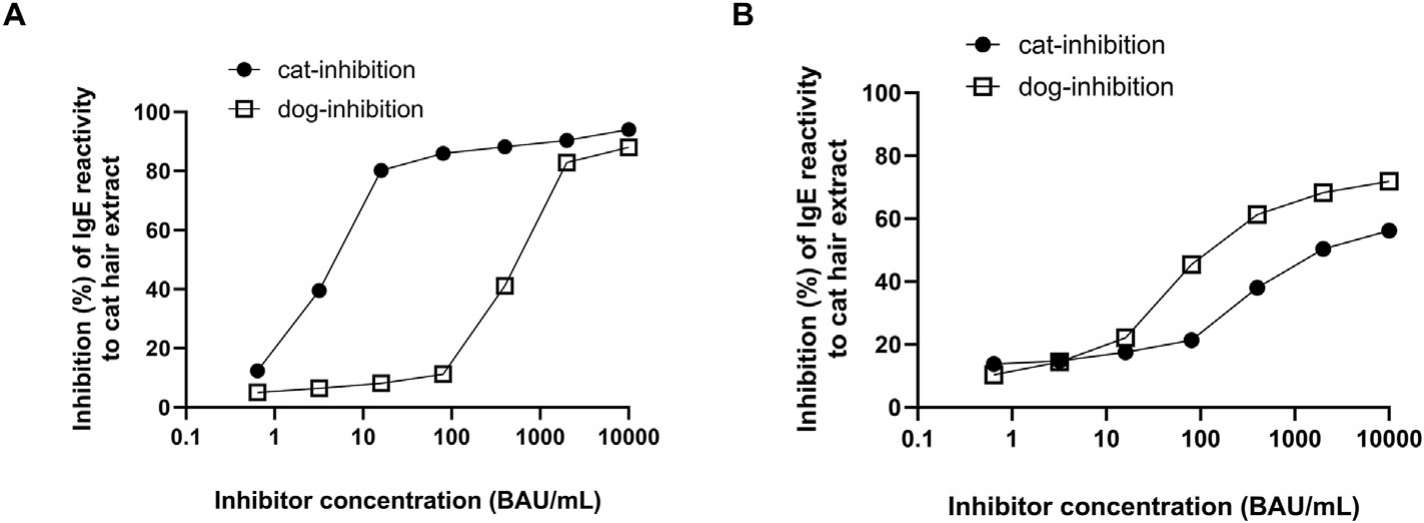
ELISA inhibition assay for cat dander extract sIgE reactivity with the corresponding inhibitors from the dog dander and cat dander. Pooled serum samples from e94 positive (A) and negative patients (B) were used

ELISA inhibition assays were conducted to assess cross-reactivity between dog and cat dander extracts using pooled sera from e94 positive (n = 7) and negative (n = 5) patients. The cat dander sIgE inhibition patterns differed markedly between the e94 positive and negative groups. In the e94 positive group, the maximum inhibition of cat dander sIgE reached 94% by cat dander and 88% by dog dander. However, the IC_50_ for a cat dander was significantly lower than for a dog dander (6.5 vs 737.9 BAU/mL). Conversely, in the e94 negative group, maximum inhibition of cat dander sIgE was 72% by dog dander and 56% by cat dander. The IC_50_ of cat dander sIgE by dog dander was significantly lower than that by cat dander in this group (172 vs 1947 BAU/mL) (Fig. 1 and Table 2).

**Table 2:** Maximum inhibition percentages and IC_50_ values of dog and cat sIgE reactivity in response to dog and cat dander extract.

Variables	Cat sIgE				Dog sIgE			
	Max inhibition (%)		IC_50_ (BAU/mL)		Max inhibition (%)		IC_50_ (BAU/mL)	
	Cat	Dog	Dog	Cat	Cat	Dog	Dog	Cat
e1+/e94+	94.0	88.0	737.9	6.5	76.1	91.5	10.6	1679
e1+/e94-	56.2	71.9	172	1947	44.6	96.8	14.7	NC

NA: not calculable

**Fig. 2:**
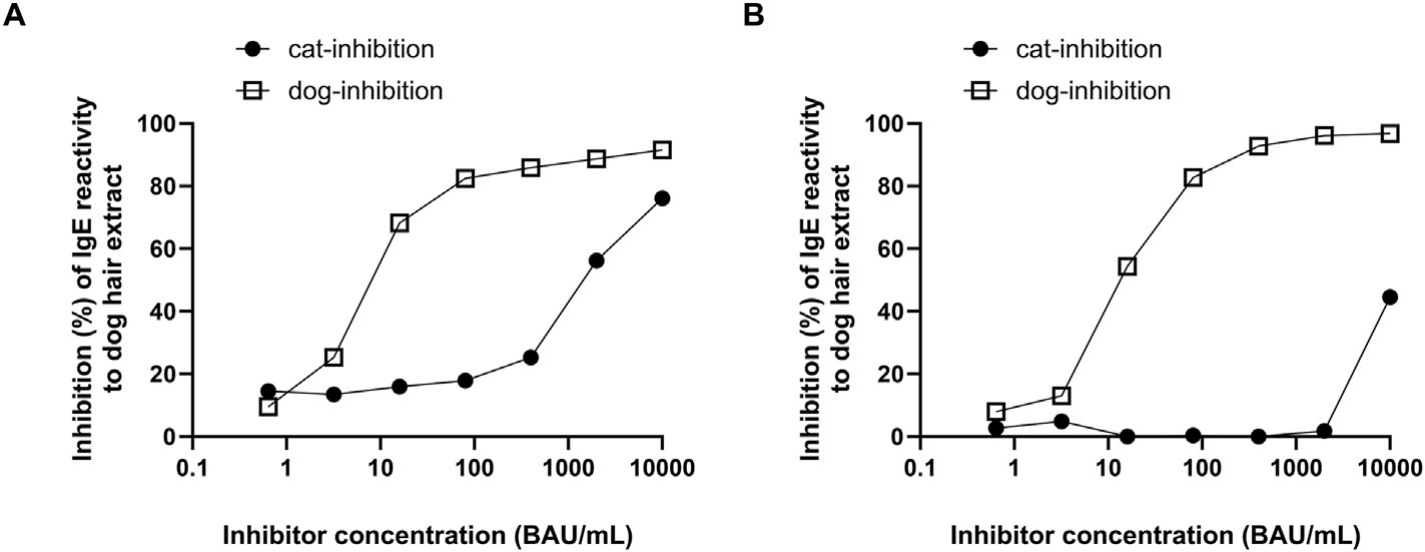
ELISA inhibition assay for dog dander extract sIgE reactivity with the corresponding inhibitors from the dog dander and cat dander. Pooled serum samples from e94 positive (A) and negative patients (B) were used

The dog dander sIgE inhibition patterns also differed significantly between e94 positive and negative groups. In the e94 positive group, maximum inhibition by dog dander was higher than that by cat dander (91.5% vs 76%). The IC_50_ for dog dander was significantly lower than that for cat dander (10.6 vs 1679 BAU/mL). In the e94 negative group, maximum inhibition by dog dander was substantially higher than that by cat dander (96.8% vs 44.6%). The IC_50_ for dog dander sIgE induced by dog dander was 14.7 BAU/mL, whereas it could not be calculated for cat dander (Fig. 2 and Table 2).

## Discussion

Our study shows that e1 positive individuals are very common (25/31) among respiratory allergy patients who exclusively own dogs. Furthermore, among the e1 positive individuals in this study, one-third (8/25) were also e94 positive. This result suggests that genuine cat sensitization is common among respiratory allergy patients who exclusively own dogs. These findings may reflect the adhesive nature of cat allergens, which can sensitize individuals even without cat ownership. Moreover, several studies have demonstrated that individuals who do not own cats exhibit reduced sensitization to lipocalins and albumin but increased sensitization to secretoglobulins (Fel d 1) compared to cat owners.^17^ Additionally, previous research by Hemmer et al indicated that cat owners have a higher rate of primary sensitization to Fel d 2, Fel d 4, and Fel d 7 compared to non-cat owners, while no significant difference was observed for sensitization to Fel d 1.^18^ These findings suggest that variations in sensitization profiles of component pet allergen may be influenced by the source of allergen exposure.

Our study suggests that a positive result of e94 could distinguish true cat sensitization from false positivity due to cross-reactivity in pet allergy patients who exclusively own dogs. Although there is some controversy,^12^ this conclusion is reasonable given that Fel d 1 is a unique allergen and has been recognized as non-cross-reactive with dog dander. Our findings revealed distinct patterns of cat sIgE inhibition between the e94 positive and negative groups. In the e94 positive group, the maximum inhibition of cat sIgE by dog dander reached 88%, but the IC_50_ by cat dander was 114 times lower than that of dog dander. If cross-reactivity were a significant issue, the IC_50_ for dog dander should be equal to or lower than that for cat dander. In contrast, the e94 negative group showed stronger inhibition of cat sIgE by dog dander than by cat dander, indicating that positive cat sIgE results are likely false positives due to cross-reactivity with dog dander (Fig. 1).

One of the primary challenges in prescribing AIT for pet allergies is the supply chain of AIT reagents, which stems from the difficulty of obtaining sufficient quantities of raw materials. To date, recombinant technology has not successfully provided sufficient quantities of major pet allergens, and total extracts from natural sources remain the only available option for AIT. Our study could prevent unnecessary prescriptions due to false positive responses, making AIT more accessible to patients with true pet allergies.

We must reiterate that the presence of e94 positivity does not exclude the presence of cross-reactivity. The patterns of cat sIgE inhibition by cat allergen were distinctive in the e94 positive group compared to the e94 negative group. Our inhibition study shows that the maximum inhibition of dog dander sIgE by cat dander was 76.1% (but the IC_50_ was 158 times higher than that of dog dander), suggesting the presence of cross-reactivity in the e94 positive group. However, our results suggest that the dog allergen extract is insufficient for suppressing cat allergy through immunotherapy in these e94 positive dog owners. Cross-reactivity is prominent in the e94 negative group. In the e94-negative group, cat sIgE was more strongly inhibited by dog dander than by cat dander, suggesting that positive cat sIgE responses are likely false positives due to cross-reactivity.

The presence of cross-reactivity between dog and cat dander is further supported by the dog dander sIgE inhibition study. In the e94 positive group, the maximum inhibition of dog dander sIgE by cat dander was 76.1%, indicating a significant degree; however, the IC_50_ of dog dander was 158 times lower. This result suggests that, although cat dander could inhibit dog dander sIgE, the cross-reactive allergenicity is weak in this e94 positive group.

The cross-reactivity of Fel d 1 with dog dander allergens remains unclear. Reininger et al revealed a Fel d 1-like allergen in dog dander, suggesting cross-reactivity between Fel d 1 and dog dander allergen.^12^ This allergen shares a high similarity to Fel d 1, indicating potential cross-reactivity. Studies have shown that recombinant Fel d 1 can inhibit dog sIgE by more than 50% in 25% of patients with Fel d 1 sIgE positive cat allergy.^12^ A study found a moderate association between sensitization to Fel d 1 and Can f_Fd1, suggesting the presence of cross-reactivity between the 2 allergens. It also revealed that sensitization to Can f_Fd1 might occur without direct exposure to dogs, indicating that cats are the predominant source of Can f_Fd1 sensitization, although the clinical significance of Can f_Fd1 remains limited.^16^

Our study may underscore that Fel d 1 is a marker of genuine cat sensitization. However, lipocalin allergens (Fel d 4, Fel d 7, Can f 1) may serve as markers for genuine pet allergy.^19^^,^^20^ Cat lipocalin allergens are cross-reactive to the counterpart of dog lipocalin allergens. Can f 1 cross-reactive to Fel d 4 and Fel d 7. Rat basophil (which express human FcεR1 receptors) releasability study demonstrated that more than 65% of sera from cat allergy patients released β-hexosaminidase to recombinant Fel d 1, Fel d 4, and Fel d 7, respectively. This study emphasizes that Fel d 4 and Fel d 7, in addition to Fel d 1, should be included in diagnostic and therapeutic regents.^21^^,^^22^ Furthermore, Can f 1 and Can f 6 are the most important major dog allergens.^16^

However, other studies have contradicted these findings, showing that nearly half of the patients sensitized to Can f 1 also had sIgE levels to Fel d 7, indicating potential cross-sensitization between Fel d 7 and Can f 1.^18^^,^^23^ Notably, among individuals positive for both Can f 1 and Fel d 7, cat owners exhibited higher levels of Fel d 7 sIgE, while dog owners showed higher levels of Can f 1 sIgE. This suggests that cat exposure can induce allergy symptoms in dog-allergic patients, and vice versa. Research indicates that cross-reactive allergens Fel d 4 and Can f 6 can inhibit IgE reactivity to each other at low competitor concentrations.^14^ Fel d 4 and Can f 6 share significant homology with Equ c 1, the primary horse allergen, suggesting the possible existence of a broader family of IgE cross-reactive lipocalins.

A previous investigation revealed that individuals who tested positive for Fel d 7 or Fel d 2 were also sensitized to Can f 1 and Can f 3, respectively.^24^ Although highly abundant, serum albumins are classified as minor allergens with a low frequency of IgE reactivity in patients allergic to the source. However, serum albumins play a crucial role in species cross-reactivity due to substantial sequence similarity among animals, with more than 70% homology with human serum albumin, and cat and dog albumins share 82% homology.^11^

Although the clinical significance of this cross-reactivity remains undetermined, our data suggest that prescribing AIT with the genuinely sensitizing allergen may be sufficient to prevent allergic symptoms caused by cross-reactive pet allergens. Consequently, Barber et al recently outlined an AIT treatment algorithm for cat and dog allergies, emphasizing the differentiation between primary sensitization and cross-reactive sIgE responses.^8^ According to this proposed algorithm, only patients sensitized to major allergens are considered eligible for AIT.

In this study, we used ImmunoCAP measurements and ELISA inhibition tests to evaluate allergen sensitization and cross-reactivity, respectively. ImmunoCAP measures sIgE under antigen excess conditions, which is optimal for diagnosing allergen sensitization. ELISA measures sIgE under antigen-antibody equilibrium conditions, which may be more suitable for evaluating cross-reactivity.^25^

In this study, we focused exclusively on e94 for molecular diagnosis, which represents both a strength and a limitation. Feline allergens such as Fel d 1, Fel d 2, and Fel d 4, and canine allergen components like Can f 1, Can f 2, Can f 3, and Can f 5 are available for sIgE testing in academic research.^24^^,^^26^^,^^27^ Measurement of these multiple molecular sIgE may provide more detailed insights into sIgE responses related to pet ownership and cross-reactivity of pet allergens. We demonstrated that a single measurement of e94 could effectively differentiate genuine sensitization from false positivity due to cross-reactivity in dog-exclusive owners. This approach is practical and easily applicable in real-world situations. Interestingly, sensitization to Fel d 1 shows no significant correlation with cat ownership.^28^^,^^29^ This finding reflects the pervasive sensitization patterns of adhesive Fel d 1 even among those without cats.

Another limitation of this study is the standardization of the allergen extracts used. We used well-standardized cat dander extract containing Fel d 1. However, the standardization of dog the dander extract is insufficient. Dog allergen extracts are generally standardized for Can f 1, Can f 5, and Can f 6; however, it remains uncertain whether other relevant dog allergens are consistently represented in the extract.

Our study shows that genuine cat sensitization is common in respiratory allergy patients who exclusively own dogs. This study is the first to demonstrate that measuring e94 can differentiate genuine cat sensitization from false positivity due to cross-reactivity in dog-exclusive owners. The presence of e94 in dog-exclusive owners suggests that Fel d 1 should be included in the AIT regimen. This finding provides important guidance for the appropriate prescription of AIT for dog allergy patients.

## Abbreviations

AIT: allergen specific immunotherapy; ICS: inhaled corticosteroids; sIgE: specific IgE.

## Availability of data and materials

The datasets in this study are available from the corresponding author on reasonable request.

## Author contributions

LL: Investigation and methodology, analysis of data, writing original draft; HAR and SYJ: Investigation and methodology; JKY, PKH and LJH: Analysis of data, critical revision of draft; PJW: Conceptualization of study, analysis of data, writing original draft, funding acquisition.

## Ethics approval

Approval for the study was obtained from the Institutional Review Boards of Yonsei University Health System (Approval no. 4-2013-0397). All participants provided written consent before enrollment.

## Consent for publication

The manuscript's publishing is approved by all of the authors.

## Funding information

This study was supported by the 10.13039/100006180Medical Device Technology Development Program (200006057, Highly sensitive three dimensional fluorescent chip for multiple allergy diagnosis) funded by the Ministry of Trade, Industry and Energy (MOTIE, Korea).

## Declaration of competing interest

The authors have no potential conflicts of interest to disclose.
